# The Development of Auditory Skills in Young Children with Mondini Dysplasia after Cochlear Implantation

**DOI:** 10.1371/journal.pone.0108079

**Published:** 2014-09-23

**Authors:** Xueqing Chen, Fei Yan, Bo Liu, Sha Liu, Ying Kong, Jun Zheng, Yongxin Li, Shusheng Gong, Demin Han, Luo Zhang

**Affiliations:** 1 Department of Otolaryngology, Head and Neck Surgery, Beijing Tongren Hospital and Beijing Institute of Otolaryngology, Capital Medical University, Beijing, China; 2 Department of Radiology, Beijing Tongren Hospital, Capital Medical University, Beijing, China; University of Salamanca- Institute for Neuroscience of Castille and Leon and Medical School, Spain

## Abstract

The aim of this study is to survey and compare the development of auditory skills in young children with Mondini dysplasia and profoundly-deaf young children with radiologically normal inner ears over a period of 3 years after cochlear implantation. A total of 545 young children (age 7 to 36 months) with prelingual, severe to profound hearing loss participated in this study. All children received cochlear implantation. Based on whether or not there was a Mondini dysplasia as diagnosed with CT scanning, the subjects were divided into 2 groups: (A) 514 young children with radiologically normal inner ears and (B) 31 young children with Mondini dysplasia. The Infant-Toddler Meaningful Auditory Integration Scale (IT-MAIS) was used to assess the children's auditory skills that include vocalization changes, spontaneous alerting to sounds in everyday living environments, and the ability to derive meaning from sounds. The assessment was performed prior to surgery and at 1, 3, 6, 9, 12, 24, and 36 months after implant device switch-on. The mean scores for overall auditory skills were not significantly different between groups A and B at pre-surgery, 1, 12, 24, and 36 months post-surgery, but were significantly different at 3, 6, and 9 months post-surgery. The mean scores for all auditory skills in children with Mondini dysplasia showed significant improvement over time. The mean scores for the three subcategories of auditory skills in children with Mondini dysplasia also showed significant differences at pre-surgery, 1, 3, 6, and 9 months, however, there were no significant differences at 12, 24, and 36 months. Overall, the auditory skills of young children with Mondini dysplasia developed rapidly after cochlear implantation, in a similar manner to that of young children with radiologically normal inner ears. Cochlear implantation is an effective intervention for young children with Mondini dysplasia.

## Introduction

The development of imaging technology has demonstrated that inner ear malformation is a common cause of congenital hearing loss in children. High-resolution computerized tomography has identified that 20% to 38% sensorineural hearing loss in children is due to cochlear malformations [1]–[4]. Mondini dysplasia, an inner ear abnormality characterized by the development of an incomplete cochlea, was first reported by Mondini in 1791 [5] based on an autopsy examination of the ears of an 8-year old boy with congenital hearing loss. Subsequent studies involving detailed evaluation using polytomography and histologic techniques have demonstrated that there is incomplete partition resulting in fusion of the middle and apical turns to form a cystic apex, whereas the basal turn of the cochlea appears to be normal [6]–[8]. Mondini dysplasia is hypothesized to be resulted from an arrest of embryogenesis at approximately the seventh gestational week as a consequence of either genetic defects or external insults [9].

Hearing levels of subjects with Mondini dysplasia may range from normal to profound deafness [8]. When profound hearing loss occurs and conventional amplification is insufficient, cochlear implantation may be an effective intervention [10]. Although patients with Mondini dysplasia were once considered as poor candidates for cochlear implantation, several studies have shown positive results for cochlear implantation in this group of patients in recent years [11]–[19]. Silverstein and colleagues first implanted a multichannel cochlear implant in a 31-year-old man with Mondini dysplasia [15]. The authors demonstrated that there were only modest improvements in objective results of Iowa test battery that comprised of closed-set tests (i.e., vowel recognition, consonant recognition, and four-choice spondee recognition) and open-set tests (i.e., monosyllabic word recognition, everyday sentence understanding, and sentence understanding without context). This adult patient did show the ability to discriminate pure-tone frequencies and environmental sounds following the implantation^15^. Subsequent studies have demonstrated that multichannel cochlear implants are useful in both children and adults with Mondini dysplasia [16]–[19]. Indeed, one study demonstrated that the sound-field thresholds following cochlear implantation of the patients with Mondini dysplasia aged from 6 to 42 years at surgery were in the region of 30–40 dB (A) [16], whereas another study showed that the aided thresholds of the patients aged from 2 to 23 years at implantation were similar to those tested in the control group with normal cochleae [17]. Moreover, cochlear implantation in children with Mondini dysplasia was shown to be successful in improving speech production and perception [18] as well as the development of listening skills [19].

Although several studies have shown a clear benefit of cochlear implantation in patients with Mondini dysplasia, there were relatively few reports of the benefits over a long period. The aim of this study is thus to evaluate the development of auditory skills in young children with Mondini dysplasia within 3 years after cochlear implantation and compare it with that in young children with radiologically normal inner ears.

## Materials and Methods

### Subjects

A total of 545 young children (338 boys and 207 girls), aged 7 months to 36 months, were enrolled into the study at Beijing Tongren Hospital, Capital Medical University, between the years 1999 and 2012. The young children presented as outpatients with severe to profound hearing loss at the Department of Otolaryngology Head and Neck Surgery and were assessed for cochlear implantation surgery.

### Study design

All young children underwent a thorough otorhinolaryngological examination and audiometric tests by experienced audiologists using behavioral audiometry and electrophysiological tests including auditory brainstem response (ABR), distortion product otoacoustic emission (DPOAE), and tympanometry. Following the examinations and tests, all children who were free of any external or middle ear pathology were assessed further for a diagnosis of prelingual, severe to profound hearing loss.

Each child with a diagnosis of prelingual severe to profound hearing loss received high-resolution computed tomography (HRCT) examination, with 1-mm contiguous slice through the temporal bone. Radiologists reviewed the CT images on a clinical picture archiving and communicating system, and examined the temporal bone for the presence of cochlear, vestibular, or semicircular canal abnormalities. The diagnosis of Mondini dysplasia was confirmed based on the presence of a shortened cochlea with only one and a half turns, incomplete interscalar septum or osseous spiral lamina between the middle and apical turns, and a fully developed basal turn. The vestibule and semicircular canals were not required to be abnormal.

Based on the absence or presence of a Mondini dysplasia, the subjects were divided into 2 groups: (A) children with a congenital sensorineural hearing loss despite having a radiologically normal inner ear and (B) children with a Mondini dysplasia. The information of the types of cochlear implant system used in the young children and other demographical information of the two groups are shown in Table 1. Four weeks after surgery the implant switch-on programming session was performed by experienced audiologists. Regular programming, assessment and intensive habilitation were followed-up for each child at specified times over a period of 3 years.

**Table 1:** Demographic and clinical characteristics of young children with radiologically normal inner ears (A) and young children with Mondini dysplasia (B).

	Group A (n = 514)	Group B (n = 31)
Type of implants		
Nucleus (n(%))	380 (74%)	25 (80%)
Medel (n(%))	52 (10%)	3 (10%)
Clarion (n(%))	82 (16%)	3 (10%)
Gender		
Male (n(%))	324 (63%)	14 (45%)
Female (n(%)	190 (37%)	17 (55%)
Age at implantation (months)		
Mean±SE	21.2±0.3	22.2±1.4
Range	7–36	9–36

The study protocol was approved by the Ethics Review Committee of Beijing Tongren Hospital and the parents or legal guardians of each child provided written informed consent prior to entry into the study.

### Measurements

With a validated translated version of the Infant-Toddler Meaningful Auditory Integration Scale (IT-MAIS) [20], the auditory skill performance of all young children was evaluated at regular intervals: pre-surgery and at 1, 3, 6, 9, 12, 24, and 36 months after switch-on programming. The IT-MAIS is a 10-question parent questionnaire that inquires about the child's spontaneous auditory behaviors in their daily life environments. The questionnaire assesses three different kinds of auditory skills: (1) vocalization changes with device use (Questions 1 and 2), (2) spontaneous alerting to sounds in everyday living environments (Questions 3 through 6), and (3) the ability to derive meaning from sounds (Questions 7 through 10). Following explanation of the questionnaire and its outcomes to the parents of children with cochlear implants, trained audiologists engaged in face-to-face sessions with the parents to elicit detailed responses to each of the questions in the IT-MAIS. The parents were asked to provide as many examples as possible of their young child's auditory skill related behaviors. The parents' responses were recorded in detail and assigned a score based on the frequency of the occurrence of target responses. Each question was assessed on a scale of 0 to 4 (0 = never, 1 = rarely, 2 = occasionally, 3 = frequently, and 4 = always) [21].

### Statistics

Statistical analyses were performed using the SPSS 13.0 statistical software. Arcsine transformation of the percentage data in this study (i.e., y = 2×sin^−1^ 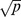, where *p* is the percentage score) was performed before the data were subjected to an analysis of variance (ANOVA) [22]. Independent sample t-test was used to determine the significance of any difference in overall auditory development between groups A and B, and also any difference in the unaided pure tone thresholds between the two groups. The significance of differences in the development of the overall and three different auditory skills in young children with Mondini dysplasia (group B) were determined using a one-way ANOVA or post-hoc analyses. A *p*-value of less than 0.05 was considered to be statistically significant.

## Results

### Subject characteristics

The demographic and clinical characteristics of the young children are shown in Table 1. The age at implantation ranged from 7–36 months with a mean age of 21.2±0.3 months. No serious cochlear implant-related or surgery-related complications were observed over the course of the study in any of the children.

### Differences in overall auditory development between groups A and B

**Figure 1:**
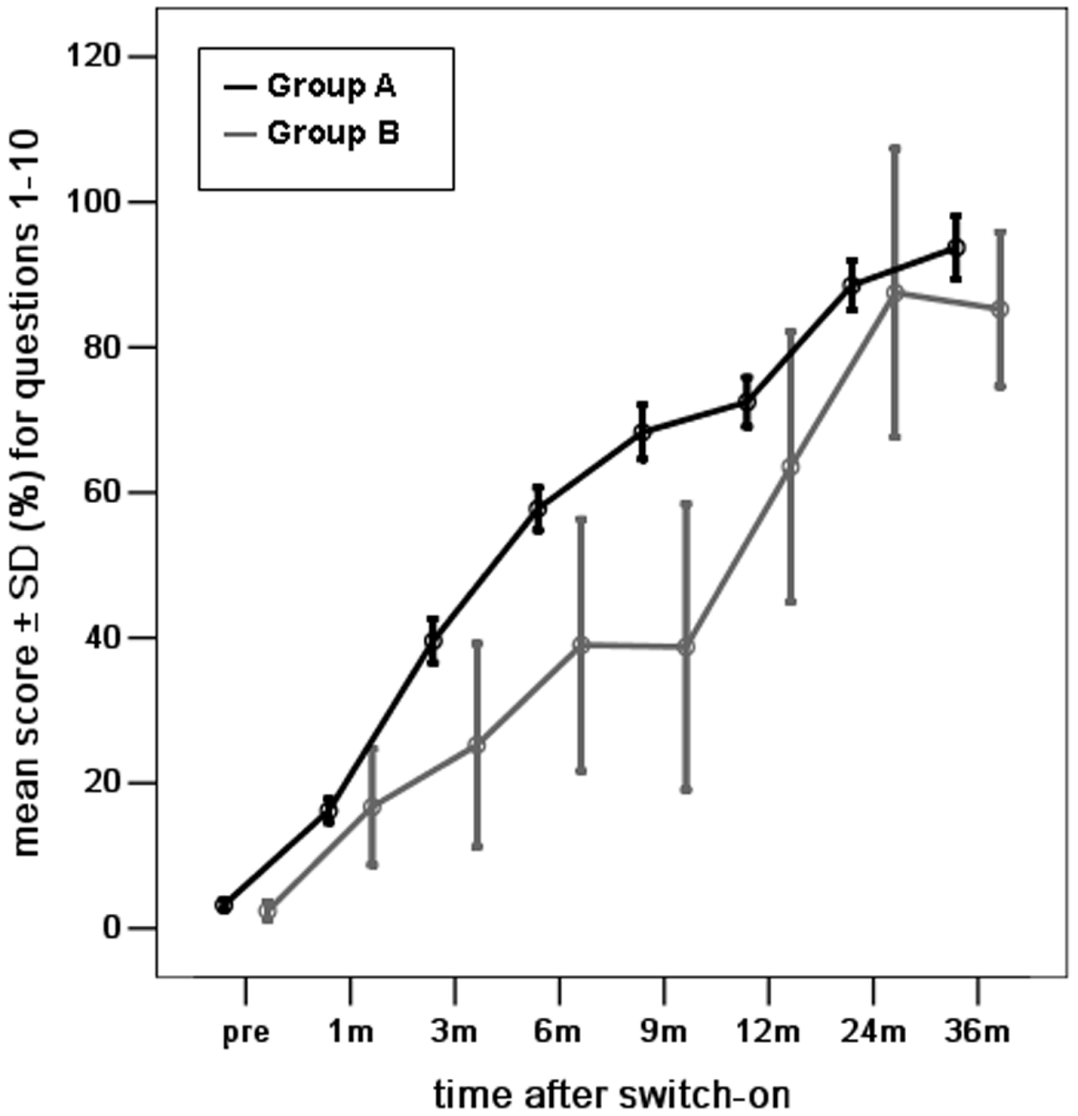
The mean scores for the development of overall auditory skills (questions 1–10) for young children with radiologically normal inner ears (Group A) and young children with Mondini dysplasia (Group B), at different intervals before and after the switch-on programming session over a period of 3 years after cochlear implantation. (score shown as percentage of total score of Questions 1–10/40×100).

The mean scores for overall auditory skills were not significantly different between groups A and B at pre-surgery and 1, 12, 24, and 36 months post-implantation surgery, however, there were significant differences at 3, 6, and 9 months post-implantation surgery (*p* <0.05) (Figure 1).

**Figure 2:**
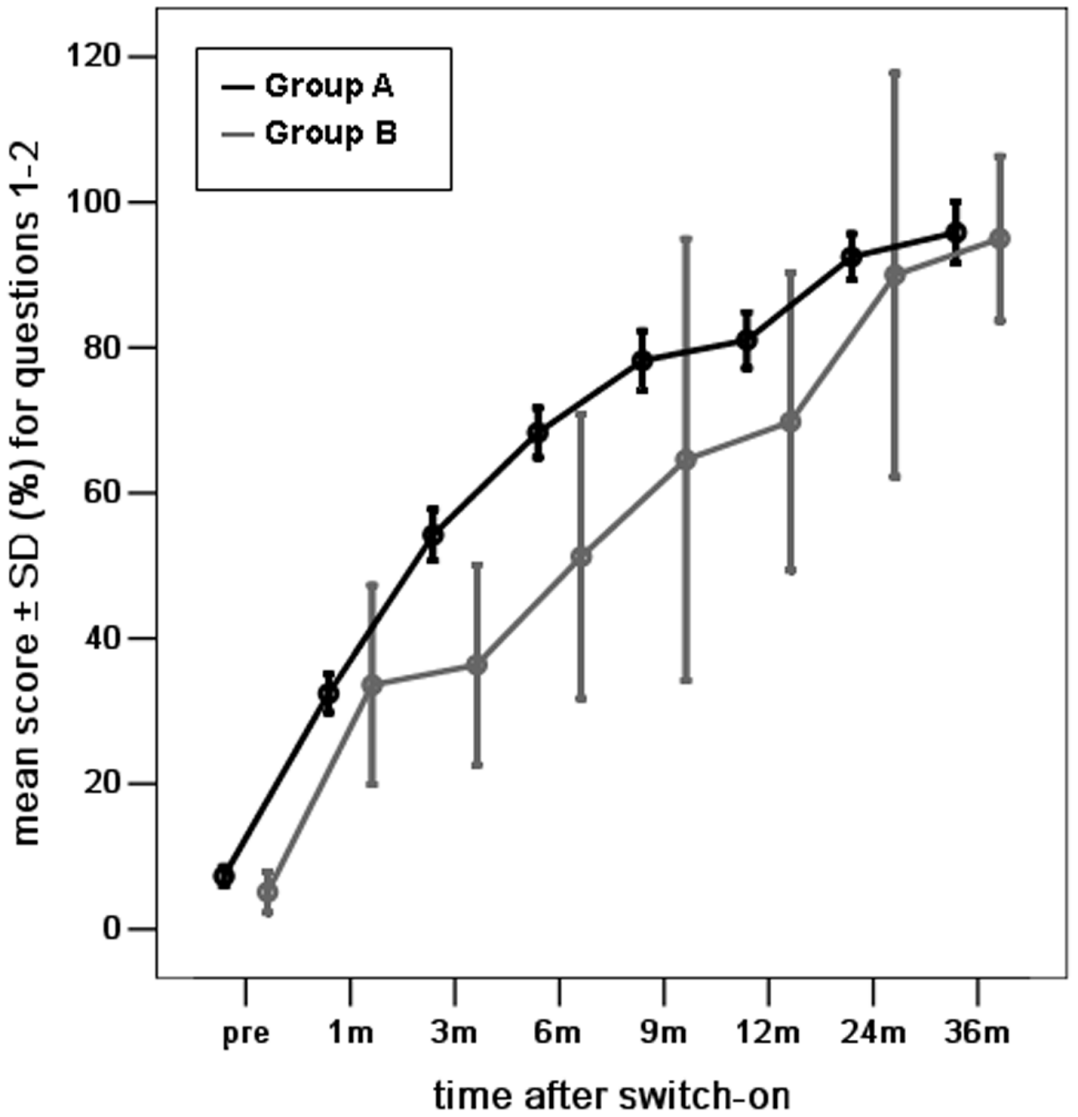
The mean scores for vocalization behaviors (questions 1–2) for young children with radiologically normal inner ears (Group A) and young children with Mondini dysplasia (Group B), at different intervals before and after the switch-on programming session over a period of 3 years after cochlear implantation. (score shown as percentage of total score of Questions 1 and 2/8×100).

**Figure 3:**
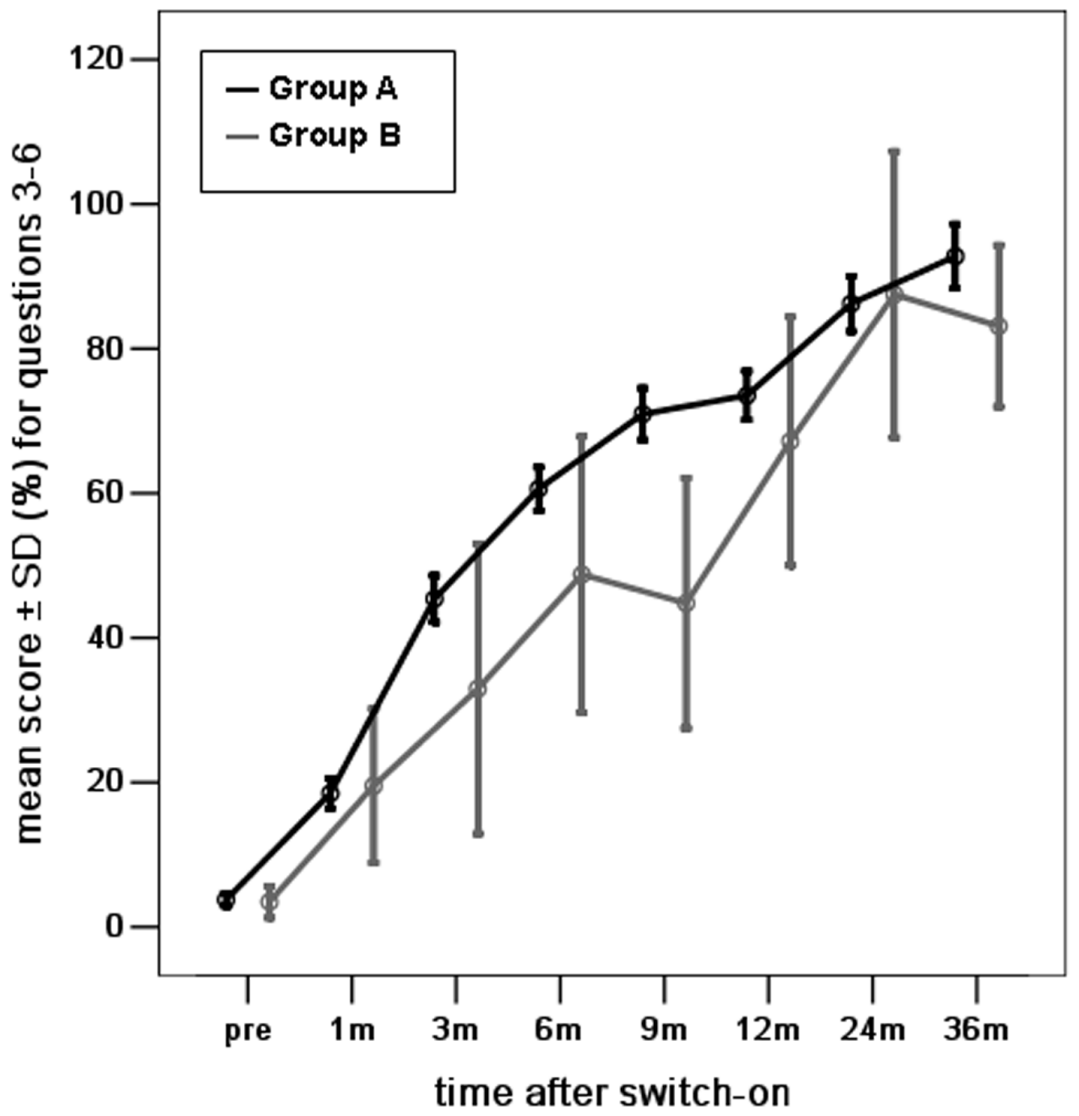
The mean scores for the development of the ability of alerting to sounds (questions 3–6) for young children with radiologically normal inner ears (Group A) and young children with Mondini dysplasia (Group B), at different intervals before and after the switch-on programming session over a period of 3 years after cochlear implantation. (score shown as percentage of total score of Questions 3, 4, 5 and 6/16×100).

**Figure 4:**
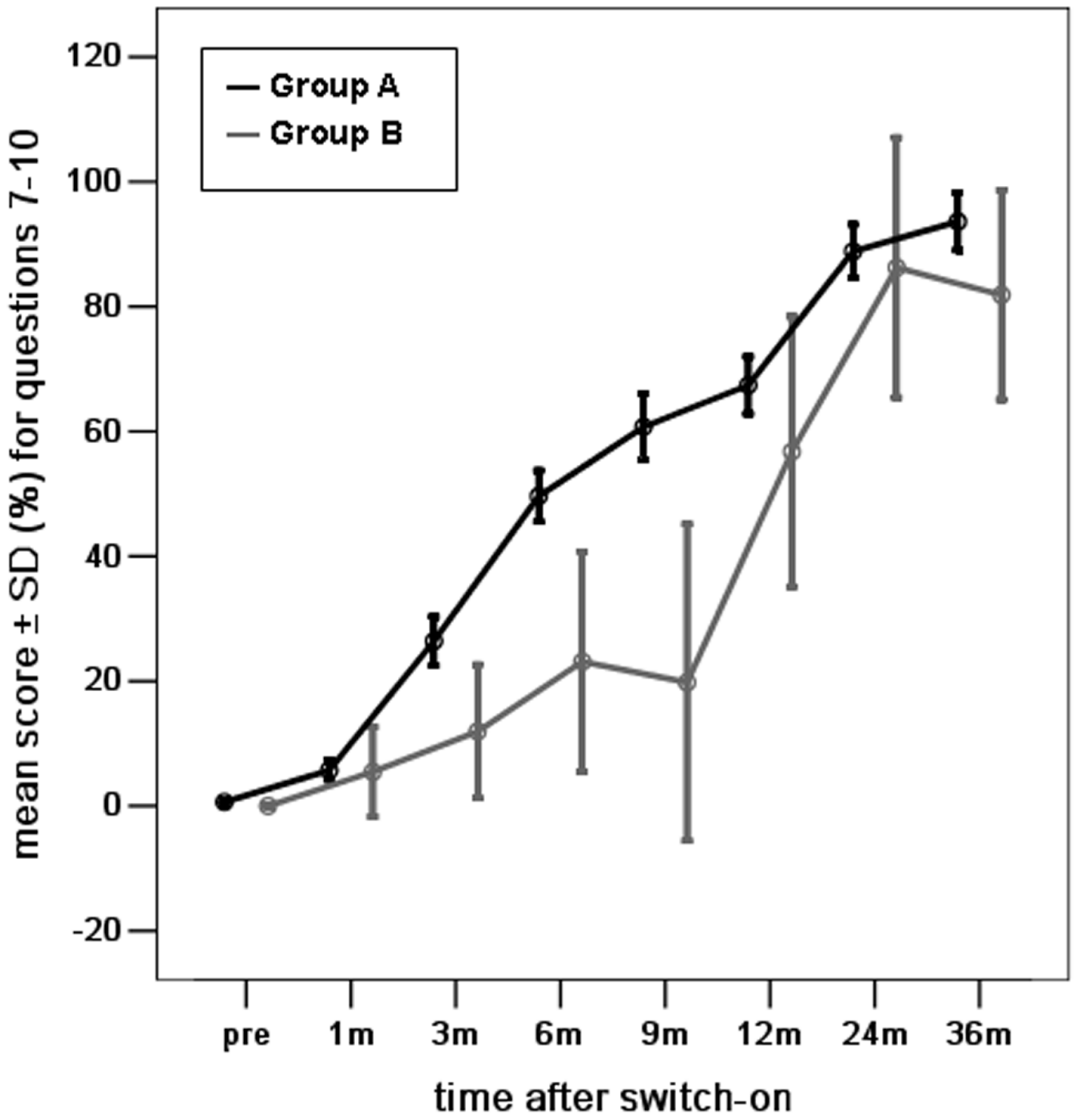
The mean scores for the development of the skill of deriving meaning from sounds (questions 7–10) for young children with radiologically normal inner ears (Group A) and young children with Mondini dysplasia (Group B), at different intervals before and after the switch-on programming session over a period of 3 years after cochlear implantation. (score shown as percentage of total score of Questions 7, 8, 9 and 10/16×100).

Next, we evaluated the auditory skills in the three subcategories (Figures 2, 3, 4). The differences in mean scores for the skill of vocalization behavior were not statistically significant between groups A and B at pre-operation and 1, 6, 9, 12, 24, and 36 months post-implantation surgery. Significant differences were observed at 3 months post-implantation surgery (*p* <0.05) (Figure 2). The mean scores for the skill of alerting to sounds were not significantly different between groups A and B at pre-surgery and 1, 3, 6, 12, and 24 months post-implantation surgery. There was a significant difference at 9, and 36 months post-implantation surgery (*p* <0.05) (Figure 3). Similarly, the differences in mean scores for the skill of deriving meaning from sounds were not statistically significant between groups A and B at 1, 3, 12, 24, and 36 months post-implantation surgery, but were significant at pre-surgery and 6, and 9 months post-implantation surgery (*p* <0.05) (Figure 4).

### The development of auditory skills in young children with Mondini dysplasia

The mean scores for all auditory skills in young children with Mondini dysplasia showed a significant improvement over time (one-way ANOVA, all *p* = 0.000) (Figures 1, 2, 3, 4). The mean scores for all auditory skills increased significantly between pre-surgery interval and each interval post-implantation surgery, as assessed by the post-hoc analyses (*p* <0.05)(Table 2). However, the scores for the skill of alerting to sounds were not significantly different between pre-surgery and 3 months post implantation surgery. The scores for the skill of deriving meaning from sounds were also not significantly different between pre-surgery and at 1, 3, 6 and 9 months post implantation surgery. The mean scores for all auditory skills increased significantly between 1 month interval and each interval after 9 months (*p* <0.05). No significant differences were noted for any of the auditory skills between 1 month and at 3, 6, and 9 months post-implantation.

**Table 2:** *P*-values of post-hoc analysis of auditory skills.

Evaluating interval	Overall auditory skills	Vocalization behavior	Alerting to sounds	Deriving meaning from sounds
pre-surgery to 1-month	0.002	0.000	0.020	0.870
pre-surgery to 3-month	0.008	0.001	0.099	0.313
pre-surgery to 6-month	0.002	0.000	0.001	0.218
pre-surgery to 9-month	0.026	0.000	0.005	0.795
pre-surgery to 12-month	0.000	0.000	0.000	0.002
pre-surgery to 24-month	0.010	0.000	0.014	0.019
pre-surgery to 36-month	0.000	0.000	0.000	0.000
1-month to 3-month	0.999	1.000	1.000	0.997
1-month to 6-month	0.314	1.000	0.167	0.772
1-month to 9-month	0.460	0.258	0.180	0.992
1-month to 12-month	0.002	0.003	0.002	0.005
1-month to 24-month	0.013	0.000	0.018	0.008
1-month to 36-month	0.000	0.000	0.000	0.000
3-month to 6-month	0.993	1.000	0.996	1.000
3-month to 9-month	0.995	0.813	1.000	1.000
3-month to 12-month	0.043	0.028	0.194	0.050
3-month to 24-month	0.015	0.000	0.039	0.008
3-month to 36-month	0.000	0.000	0.007	0.000
6-month to 9-month	1.000	1.000	1.000	1.000
6-month to 12-month	0.704	1.000	0.927	0.533
6-month to 24-month	0.061	0.023	0.235	0.016
6-month to 36-month	0.003	0.000	0.067	0.002
9-month to 12-month	0.736	1.000	0.644	0.661
9-month to 24-month	0.074	0.695	0.178	0.034
9-month to 36-month	0.008	0.053	0.019	0.017
12-month to 24-month	0.754	1.000	0.973	0.573
12-month to 36-month	0.481	0.064	0.994	0.470
24-month to 36-month	1.000	1.000	1.000	1.000

A *p*-value of less than 0.05 is considered statistically significant.

Comparison of scores at time intervals between 3 to 9 months indicated no significant differences for all auditory skills between 3 months and at 6 and 9 months. The differences were statistically significant in the mean scores for all auditory skills between 3 months and those after 9 months (*p* <0.05), except for the scores between 3 and 12 months interval post implantation surgery for the skills of alerting to sounds and deriving meaning from sounds. No significant differences were found for the scores of all auditory skills between 6 months and at 9, 12, and 24 months, except between 6 and 24 months interval post-implantation surgery for the skills of vocalization behavior and deriving meaning from sounds (*p* <0.05). The differences were statistically significant in the mean scores for all auditory skills between 6 and 36 months (*p* <0.05). However, no significant difference was noted for the skill of alerting to sounds during this time interval.

Similarly, comparison of the scores for time intervals between 9 to 36 months showed no significant differences for all auditory skills between 9 months and 12 or 24 months, except for the scores between 9 and 24 months for the skill of deriving meaning from sounds (*p* <0.05). The mean scores for all auditory skills increased significantly between 9 and 36 months (*p* <0.05), except for the skill of vocalization behavior during this time interval. No significant differences were noted for the mean scores for all auditory skills between 12 months and any intervals after 12 months. The mean scores for all auditory skills also indicated no significant differences between 24 and 36 months.

**Figure 5:**
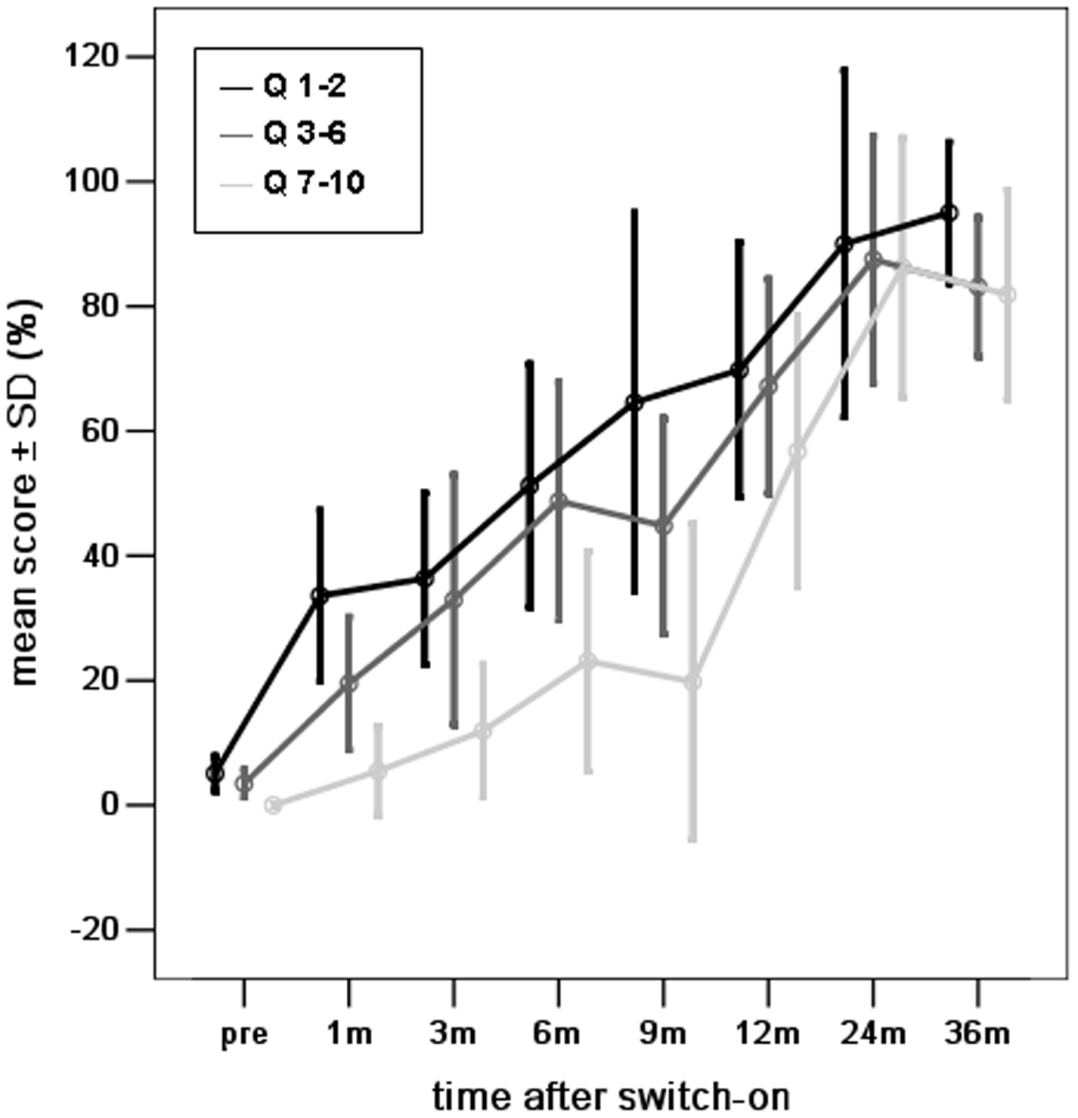
The mean scores for vocalization behaviors (questions 1–2), the ability of alerting to sounds (questions 3–6), and the skill of deriving meaning from sounds (questions 7–10) for young children with Mondini dysplasia (Group B), at different intervals before and after the switch-on programming session over a period of 3 years after cochlear implantation.

The mean scores for the three individual auditory skills in young children with Mondini dysplasia showed significant differences at pre-surgery, 1, 3, 6, and 9 months (all *p*<0.05), but not at any other study periods 12 months onwards (Figure 5).

### The differences in unaided pure tone thresholds between groups A and B

Table 3 shows the mean unaided pure tone thresholds at octave frequencies from 0.25 to 4 kHz for groups A and B before surgery. There were significant differences between the two groups at 2 kHz for either ear and at 1 kHz for the right ear (*p* <0.05), but not at any other octave frequencies tested.

**Table 3:** Unaided pure tone thresholds of young children with radiologically normal inner ears (Group A) and young children with Mondini dysplasia (Group B).

	Left ear					Right ear				
Frequency(kHz)	0.25	0.5	1	2	4	0.25	0.5	1	2	4
Group A										
Mean (dB HL)	99	105	109	112	113	100	105	109	112	113
SD (dB HL)	13	11	9	10	10	12	12	10	11	11
Group B										
Mean (dB HL)	100	108	104	105	111	98	103	104	104	107
SD (dB HL)	18	12	12	11	11	17	13	12	11	12

## Discussion

This study showed that the auditory skills of young children with Mondini dysplasia developed rapidly over a period of 3 years after cochlear implantation, in a similar manner to those of young children with radiologically normal inner ears. Otte et al [23] suggested that there are around 36,000 spiral ganglion cells in subjects with a normal cochlea in the first decade of their lives and that at least 10,000 ganglion cells with no less than 3000 located in the apical 10 mm of the organ of Corti are necessary for speech discrimination. Other researchers suggested that the spiral ganglion cell is the vital neural element required for electrical stimulation through a cochlear prosthesis [24], [25] and that a smaller number of ganglion cells may be necessary when an electrical stimulation is applied, compared to a larger number of ganglion cells being necessary when an acoustic stimulation is presented. Linthicum et al. [26] reported that two cochlear implant users with normal bony cochleae and average scores on auditory testing had only 3,212 and 3,376 surviving spiral ganglion cells, respectively. Another histological study by Schmidt [27] assessing the number of cochlear neuronal cells in ears of 20 subjects with hearing loss caused by developmental defects demonstrated that the numbers of spiral ganglion cells in Mondini dysplasia ranged from 7,677 in one case to 16,110 in another case. Although the minimum number of spiral ganglion cells necessary for response to electrical stimulation has not been ascertained, the number of cells noted in subjects with even severe Mondini dysplasia is probably large enough to trigger neural responses by electrical stimulation from a cochlear implant system [26]. In this context, this may explain why the mean scores of auditory skills were not significantly different between the two groups of subjects investigated in the current study after 1 year of implant use. Alternatively, this lack of a significant difference between the two groups may also be a consequence of similar mean unaided pure tone thresholds at each octave frequency from 0.25 to 4 kHz before cochlear implantation in the young children from both groups; which allowed the children with Mondini dysplasia to develop appropriate auditory skills relative to the young children with radiologically normal inner ears.

Furthermore, as all young children were recommended to attend regular mapping sessions and effective speech and hearing rehabilitation programs after cochlear implantation, these programs might have helped all the young children with Mondini dysplasia develop their auditory skills quite well and at similar rates when compared to the young children with radiologically normal inner ears [28], [29]. Previous study has indicated that early implantation provides better opportunity for children with profound hearing loss to acquire communication skills [21]. Tone recognition and production were consistently correlated with age at implantation [30], [31]. Pediatric patients should be implanted early to receive maximum benefit from a cochlear implant [30]–[32]. With the application of universal newborn hearing screening (UNHS) and the development in hearing diagnosis technology, it has become possible to identify children with congenital hearing loss at an early age and thus helped clinicians to diagnose and treat Mondini dysplasia patients more easily by cochlear implant surgery. This has in turn provided children with Mondini dysplasia a better opportunity to be able to develop auditory skills at an early age.

A study by Kim and colleagues [33] has indicated that although children with inner ear malformation receive considerable benefit from cochlear implants over a long period, there may be a slight delay in the development of auditory skills in these children at the early stages after cochlear implantation surgery. Our findings are in accordance with the findings of Kim and colleagues [33] in that we observed a relatively slow development of auditory skills, especially in the first year after cochlear implantation surgery in young children with Mondini dysplasia. Our findings further demonstrated that despite this delayed development in the auditory skills of children with Mondini dysplasia, they catch up with those with radiologically normal inner ears after 1 year of implant use, and show similar overall auditory skills as well as the three individual subcategories of auditory skills within the first two years after cochlear implantation. Our findings are also in accordance with those of Kim and colleagues [33] who demonstrated that the Phonetically Balanced Kindergarten test for phonemes was not statistically significant between children with inner ear malformations and those with radiologically normal inner ears at 2 years after cochlear implantation. Furthermore, the findings from the current study in children with Mondini dysplasia are consistent with our previous findings [32], [34] in children with radiologically normal inner ears and large vestibular aqueduct syndrome (LVAS) that development of the three individual subcategories of auditory skills follows a similar pattern. In particular, the improvement of vocalization behavior was superior to the other two subcategories of auditory skills, especially within the first year after cochlear implantation, and the ability of deriving meaning from sounds was the slowest developed skill.

In conclusion, the findings of this study confirm that cochlear implantation is an effective intervention for young children with Mondini dysplasia, when the amplification of optimal hearing aids is insufficient. These findings further indicate that young children with Mondini dysplasia require a slightly longer time to attain appropriate auditory skills than those with radiologically normal inner ears and thus suggest that more attention should be paid to their training during the first year post implantation. Overall, these data provide a basic time-frame for speech and hearing habilitation programs for young children with Mondini dysplasia following cochlear implantation surgery and highlight the importance of early implantation in those children.
